# Nothing a Hot Bath Won't Cure: Infection Rates of Amphibian Chytrid Fungus Correlate Negatively with Water Temperature under Natural Field Settings

**DOI:** 10.1371/journal.pone.0028444

**Published:** 2011-12-21

**Authors:** Matthew J. Forrest, Martin A. Schlaepfer

**Affiliations:** 1 Center for Marine Biodiversity and Conservation, Scripps Institution of Oceanography, La Jolla, California, United States of America; 2 State University of New York, College of Environmental Science and Forestry, Syracuse, New York, United States of America; 3 INRA-SCRIBE, Rennes, France; University of Minnesota, United States of America

## Abstract

Dramatic declines and extinctions of amphibian populations throughout the world have been associated with chytridiomycosis, an infectious disease caused by the pathogenic chytrid fungus *Batrachochytrium dendrobatidis* (*Bd*). Previous studies indicated that *Bd* prevalence correlates with cooler temperatures in the field, and laboratory experiments have demonstrated that *Bd* ceases growth at temperatures above 28°C. Here we investigate how small-scale variations in water temperature correlate with *Bd* prevalence in the wild. We sampled 221 amphibians, including 201 lowland leopard frogs (*Rana* [*Lithobates*] *yavapaiensis*), from 12 sites in Arizona, USA, and tested them for *Bd*. Amphibians were encountered in microhabitats that exhibited a wide range of water temperatures (10–50°C), including several geothermal water sources. There was a strong inverse correlation between the water temperature in which lowland leopard frogs were captured and *Bd* prevalence, even after taking into account the influence of year, season, and host size. In locations where *Bd* was known to be present, the prevalence of *Bd* infections dropped from 75–100% in water <15°C, to less than 10% in water >30°C. A strong inverse correlation between *Bd* infection status and water temperature was also observed within sites. Our findings suggest that microhabitats where water temperatures exceed 30°C provide lowland leopard frogs with significant protection from *Bd*, which could have important implications for disease dynamics, as well as management applications.

*There must be quite a few things a hot bath won't cure, but I don't know many of them* - Sylvia Plath, “The Bell Jar” (1963).

## Introduction

Chytridiomycosis, an infectious disease caused by the pathogenic chytrid fungus *Batrachochytrium dendrobatidis* (*Bd*), is a primary factor in worldwide amphibian declines and species extinctions [1], [2]. *Bd* belongs to a group of virulent multi-host pathogens that have had profound effects on entire communities and ecosystems [3]. In fact, *Bd* has been called “possibly the most deadly invasive species on the planet (excluding humans)” [4]. Although amphibian susceptibility to *Bd* and chytridiomycosis is species-specific, environmental conditions also appear to modify host-disease dynamics [2]. The prevalence of *Bd* (i.e. the proportion of infected animals) and the virulence of chytridiomycosis are particularly influenced by temperature [5]. Field studies conducted in disparate geographic regions show *Bd* infections are generally more severe in winter months, and when hosts are found in cooler temperatures [6]–[9].

In the laboratory *Bd* cultures grew and reproduced at temperatures between 4–25°C, with maximal growth at 17–25°C, but growth ceased at temperatures above 28°C [10]. Incubation of *Bd* cultures at 30°C for 8 days killed 50% of colonies [10], and 100% mortality occurred within 96 hours at 32°C and within 4 hours at 37°C [11]. Similarly, *Bd* does not persist in amphibian hosts above certain temperature thresholds. In laboratory experiments, short-term exposure to temperatures between 27 and 37°C successfully cleared *Bd* infections from five species of adult frogs with no reported side effects [7], [12]–[14]. Although *Bd* is susceptible to certain antifungal agents when tested in vitro, there are few proven methods for clearing infections in adult amphibians, and acute drug toxicity can be a problem for tadpoles and juveniles [15]–[17]. Therefore, heat treatments may be a superior alternative to currently available antifungal drugs for captive animals infected with *Bd* [14], [17]. However, whether amphibians in the wild can also be cleared of *Bd* by short-term exposure to elevated temperatures remains unknown.

Several species of leopard frogs naturally inhabit geothermal ecosystems in the southwestern United States, including endangered and threatened species. For example, all naturally occurring populations of the relict leopard frog (*Rana* [*Lithobates*] *onca*) are now associated with perennial geothermal springs in Nevada with source temperatures exceeding 30°C [18], and geothermal spring sites in New Mexico are particularly important breeding habitats for the threatened Chiricahua leopard frog (*Rana* [*Lithobates*] *chiricahuensi*s) [19]. Observations from a previous study indicated that *Bd* was less prevalent in frogs inhabiting geothermal waters in Arizona, USA [20]. Consequently, we tested whether *Bd* is negatively associated with water temperature by sampling amphibian populations from several geothermal ecosystems, as well as non-geothermal sites.

All seven native Arizona ranid species have experienced significant population declines and local extinctions [21], and chytridiomycosis appears to be an important contributory factor–particularly during the winter months [6], [22]. Lowland leopard frogs (*Rana* [*Lithobates*] *yavapaiensis*) have been extirpated from nearly half of their historic geographic range [21], and populations continue to decline or disappear from additional sites [23].

Identifying environments and climactic conditions that provide natural refuges from *Bd* will benefit imperiled amphibian populations [24], [25], and may provide some susceptible species with opportunities to evolve evolutionary responses to the pathogen [26]. Information on the environmental limitations of *Bd* in the wild is critical to the conservation of amphibians affected by this disease [27], yet a clear understanding of how temperature modulates host-disease dynamics in the field has remained elusive to date [28], [29]. Geothermal settings provide unique opportunities to examine the effects of a wide range of environmental temperature on chytridiomycosis host-disease dynamics in wild amphibian populations. To our knowledge, this study is the first to utilize steep natural temperature gradients in field settings, and measure water temperature at the precise time and place where each amphibian was captured, thereby providing valuable information collected at a fine spatial scale to help elucidate the thermal restrictions of *Bd* under natural field conditions.

## Results

**Figure 1:**
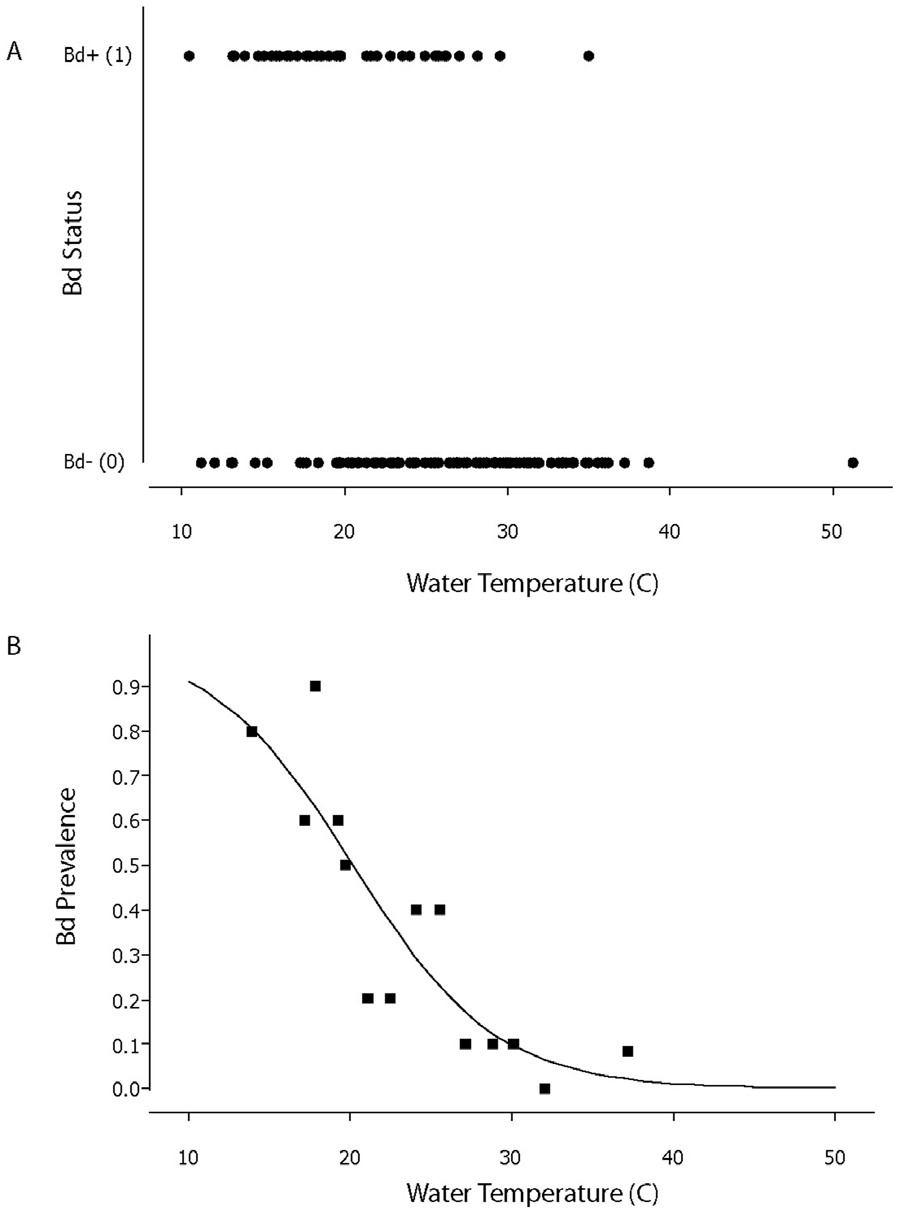
Occurrence of *Bd* in lowland leopard frogs as a function of water temperature. A) Presence (1) and absence (0) of *Batrachochytrium dendrobatidis* (*Bd*) in lowland leopard frogs *Rana* (*Lithobates*) *yavapaiensis* as a function of water temperature across all years and sites. N = 50 *Bd*+ individuals and N = 151 *Bd*−. B) Occurrence of *Bd* in lowland leopard frogs as a function of water temperature, excluding four sites where *Bd* was not detected (see Table 1 for details). Each point represents the fraction of frogs infected and mean water temperature of groups of 10–12 individuals (ranked by water temperature, then binned). Logistic equation: y = 1/(1+e^−z^) where z = 4.56 - temp*0.226 (coefficients extracted from univariate analysis). Total sample size from sites with *Bd*, N = 148.

We sampled 221 post-metamorphic anurans belonging to five species, of which the lowland leopard frog (n = 201) was the most common (Table 1). The primary result was a significant negative association between *Bd* infection status of lowland leopard frogs and water temperature. Overall, the temperature of the water in which we captured *Bd*-positive (*Bd*+) individuals (mean = 19.8±0.67°C, n = 50) was significantly cooler than for *Bd*-negative (*Bd*−) individuals (mean = 25.8±0.50°C, n = 151; 2-sample t-test: df = 108, t = 7.1, p<0.001; Fig. 1A). The negative association between water temperature and *Bd* infection status was apparent in the univariate test, but also after accounting for the influence of other significant factors such as Distance to Solstice (i.e., seasonality) and Year (Table 2).

**Table 1:** Sites sampled, Latitude and Longitude (Datum = WGS 84), dates sampled, range of water temperatures measured during sampling, species sampled, number of individuals, and *Bd* prevalence (percent infected) per species sampled.

Location	Dates Sampled	H_2_O Temp (°C)	Species Sampled (n)	Species *Bd* Prevalence
**Aravaipa Creek**	Oct 2004	17.7	*Rana yavapaiensis* (11)	0.64
N32 52.725	May 2009	23.0		
W110 23.767	Feb 2010	10.5		
**BHP Hotwell** *	Feb 2010	24.3–28.1	*R. yavapaiensis* (7)	0.00
N32 37.961				
W110 33.537				
**Dankworth Pond** *	Mar 2009	34.1	*Rana catesbeiana* (1)	0.00
N32 43.279				
W109 42.089				
**El Dorado Hot Spring** *	May 2009	36.5–34.1	*R. yavapaiensis* (18)	0.00
N33 29.588			*Bufo alvarius* (1)	0.00
W112 56.442				
**Essence of Tranquility** *	Mar 2009	30.7	*R. catesbeiana* (1)	0.00
N32 45.480				
W109 43.510				
**Hassayampa – TNC**	Oct 2004	19.7	*R. yavapaiensis* (8)	0.63
N33 55.838			*R. catesbeiana* (2)	0.00
W112 41.520				
**Hassayampa Preserve**	Oct 2004	19.7	*R. yavapaiensis* (6)	0.00
N34 02.472				
W112 42.235				
**San Pedro River**	Mar 2009	16.0	*R. yavapaiensis* (1)	1.00
N32 55.511			*R. catesbeiana* (3)	0.66
W110 44.489				
**Mammoth Hot Well** *	Oct 2004	22.4–37.2	*R. yavapaiensis* (60)	0.21
N32 41.660	Mar 2009	18.4–36.2	*R. catesbeiana* (10)	0.00
W110 37.341	Feb 2010	15.1–33.4	*Bufo woodhousii* (1)	0.00
**Markham Creek** 1	May 2009	22.4–24.0	*R. yavapaiensis* (29)	0.00
	Feb 2010	11.2–14.5	*Hyla arenicolor* (1)	0.00
**Muleshoe Hot Spring** *	Aug 2004	22.8–51.2	*R. yavapaiensis* (27)	0.19
N32 20.229	Mar 2009	22.0–27.0		
W110 14.331	Feb 2010	13.1–30.0		
**Secret Spring** *	Aug 2004	21.6–23.6	*R. yavapaiensis* (34)	0.56
N32 20.395	Mar 2009	13.9–38.7		
W110 14.613	Feb 2010	16.6–18.3		

*Indicates presence of a geothermal source at location.

1 Coordinates excluded at the request of U.S. Bureau of Land Management (Safford, AZ).

**Table 2:** Logistic binary regression of infection status (0 = not infected; 1 = infected with *Batrachochytrium dendrobatidis*) of lowland leopard frogs *Rana* (*Lithobates*) *yavapaiensis* (N = 198; 3 missing SVL).

Predictor^1^	Coefficient	Standard Error Coefficient	Z	P	Odds Ratio	Lower 95% CI	Upper 95% CI
Constant	−1.02834	2.18103	−0.47	0.637			
Water Temp	−0.188474	0.0424268	−4.44	0.000	0.83	0.76	0.90
Dist Solstice	0.0648780	0.0155885	4.16	0.000	1.07	1.03	1.10
SVL (mm)	−0.0240867	0.0188217	−1.28	0.201	0.98	0.94	1.01
Year							
2009	0.310455	0.579158	0.54	0.592	1.36	0.44	4.24
2010	−3.80550	0.865731	−4.40	0.000	0.02	0.00	0.12

Log-Likelihood = −70.328.

Test that all slopes are zero: G = 83,12, DF = 5, P-Value = 0.000.

1 Years 2009 and 2010 contrasted with 2004. Dist. Solstice = Distance to Solstice, the absolute difference in days between the sampling date and June 21^st^ (used to capture seasonal variation). SVL = Snout-Vent Length.

To control for a possible absence of *Bd* from certain sites (Table 1) we repeated the binomial logistic regression analysis excluding samples from Markham Creek, Upper Hassyampa, El Dorado Hotspring and BHP Hotwell, where more than one individual was tested, and *Bd* had not been detected. The analysis on the restricted dataset (n = 148) yielded a qualitatively identical result for the binomial regression, with *Bd* prevalence declining sharply from 100% at 10–15°C until 30°C, where it approaches zero (Fig. 1B). Our subsequent analyses include data from all sites because our samples sizes at any particular site were too small to be confident that *Bd* was truly absent (Table 1).

Distance to Solstice and Year were also significantly associated with *Bd* prevalence (Table 2). The positive association between *Bd* prevalence and Distance to Solstice illustrates that individuals were more likely to be *Bd*+ during early spring and late fall sampling than during the summer, even after taking into account water temperatures (Table 2). *Bd* prevalence also varied between years, with the prevalence in 2009 being significantly lower than in 2004 and 2010 once the time of year was taken into account (Table 2). Because slightly different methods for assaying *Bd* were used in the 2004 study, we repeated all analyses using only the 2009 and 2010 data and found qualitatively similar results.

Size of host (SVL) was not significantly associated with *Bd* prevalence in the binomial regression (Table 2). Nevertheless, other results suggest that the relationship between *Bd* and host may change with size. We observed that seven of the eight *Bd*+ individuals that were captured in waters warmer than 25°C were juveniles (≤50 mm SVL). Furthermore, the mean water temperature in which *Bd*+ juveniles were captured (21.6±1.31°C, n = 20) was marginally higher than that of *Bd*+ adults (18.7±0.64°C, n = 30; t-test: df = 28, t = 2.00, p = 0.056). Our results suggest that juveniles and adults may differ in their susceptibility to *Bd* or in their behavior once infected.

*Bd* prevalence clearly varies between sites (Table 1), and we wanted to ensure that the correlation between water temperature and *Bd* prevalence was not confounded by site characteristics. When Site was included in the binomial logistic model, Water Temperature remained a significant factor, but certain site-specific coefficients could not be estimated (likely because of small sample sizes). We therefore undertook an additional analysis in which we compared AICc values (Akaike information criterion corrected for finite sample size) of two logistic binomial models with Sex, SVL, Year and Site as factors, but where Water Temperature was present only in the first model. The much lower AICc value (139.1 vs. 158.3) associated with the first model, which included Water Temperature, strongly suggests a significant correlation between *Bd* prevalence and water temperature, even when accounting for variation in *Bd* between sites.

**Figure 2:**
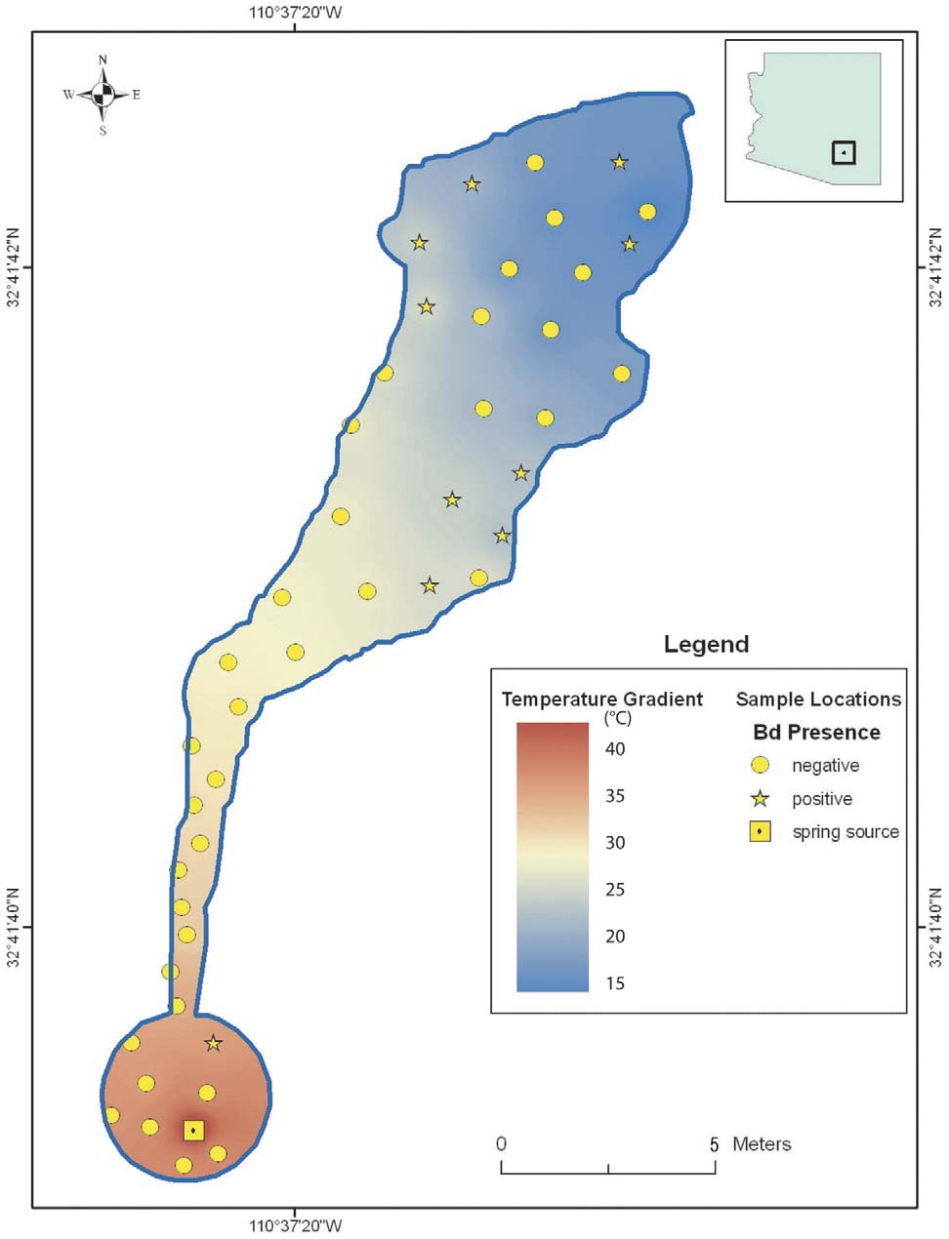
Schematic of Mammoth Hot Well showing approximate locations of amphibians and water temperatures. Symbols show approximate locations of lowland leopard frogs (redrawn from field notes) sampled in March 2009, their *Bd* infection status, and the range of measured water temperatures.

**Figure 3:**
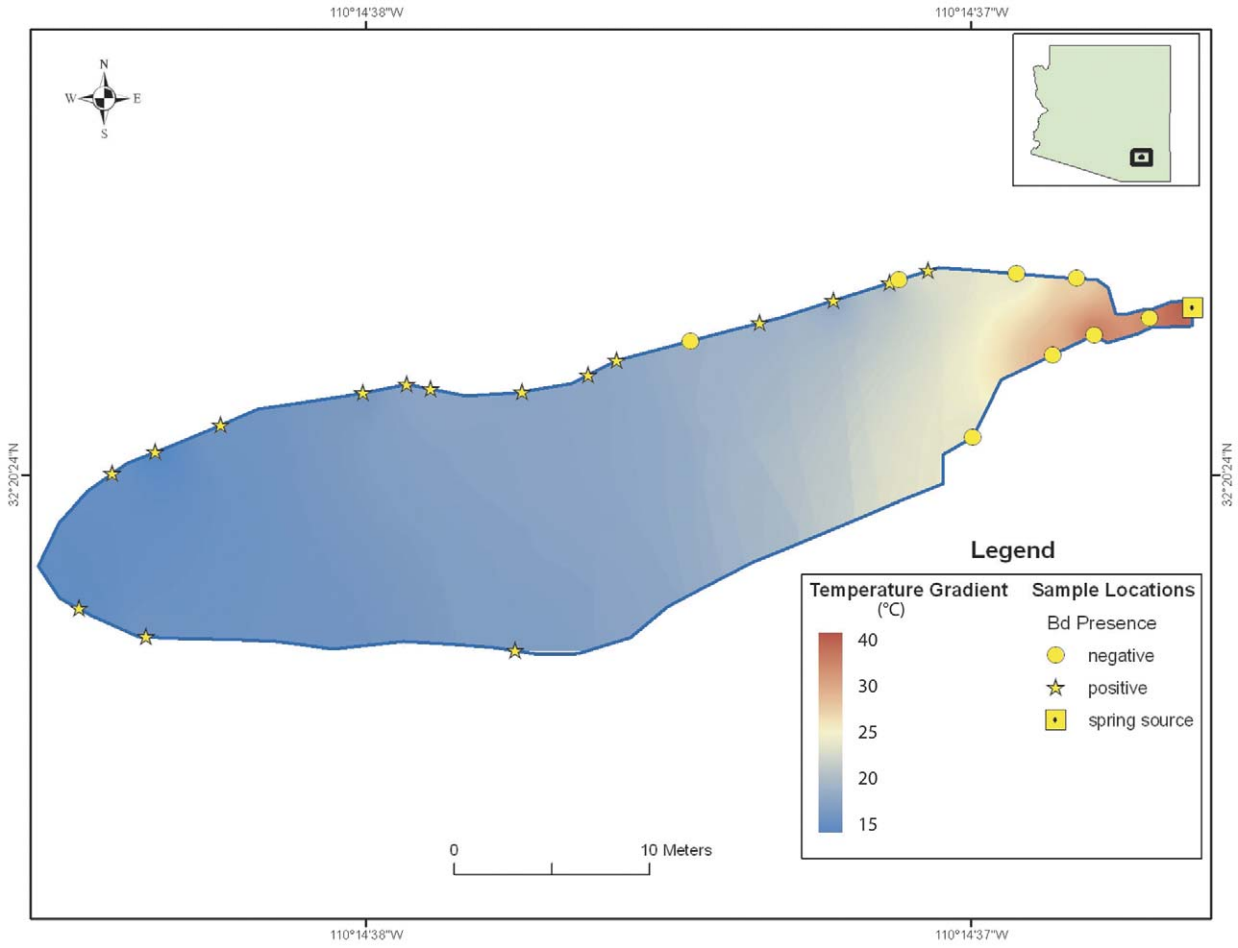
Schematic of Secret Spring showing approximate locations of amphibians and water temperatures. Symbols show approximate locations of lowland leopard frogs (redrawn from field notes) sampled in March 2009, their *Bd* infection status, and the range of measured water temperatures.

The negative relationship between *Bd* prevalence and water temperature was also apparent within the Secret Spring and Mammoth Hot Well sites, where sampling covered a wide range of water temperatures from the relatively constant, elevated temperatures at their sources to more variable, ambient temperatures in the distal portions of the ecosystems. *Bd* was detected at these sites, but more commonly in individuals located in cooler waters. At both sites, the prevalence of *Bd* was significantly lower in water temperatures of 30°C and above (Secret Spring: 19/30 Bd+ in water <30°C, and 0/4 Bd+ in water ≥30°C; two-tailed Fisher's exact test; p = 0.0294; Mammoth Hot Well: 13/45 Bd+ in water <30°C, and 1/26 Bd+ in water ≥30°C; two-tailed Fisher's exact test p = 0.0124). In 2009 we mapped the location of each individual captured at these two locations. Eight of the nine *Bd*+ individuals (89%) at Mammoth Hot Well were found in waters cooler than 27°C (Fig. 2), and all 15 *Bd*+ individuals (100%) at Secret Spring were found in waters cooler than 20°C (Fig. 3).

We also tested 20 individuals of four other species: 17 American bullfrogs (*Rana* [*Lithobates*] *catesbeiana*), 1 canyon tree frog (*Hyla arenicolor*), 1 Woodhouse's toad (*Bufo* [*Anaxyrus*] *woodhousii woodhousii*), and 1 Sonoran toad (*Bufo* [*Incilius*] *alvarius*) (Table 1). All were *Bd*−, with the exception of two American bullfrogs captured at 16°C.

## Discussion

This study provides evidence that the probability that a lowland leopard frog is infected with *Bd* is significantly negatively correlated with water temperature at the point of capture (Fig. 1A and 1B). Previous field studies documented a correlation between *Bd* prevalence and seasonality, with higher infection rates during cool seasons [6]–[9], [28]. In this study, we documented that the inverse relationship between water temperature and *Bd* prevalence also holds true within seasons. Furthermore, the association between temperature and *Bd* prevalence was apparent even within bodies of water, with *Bd*+ frogs largely absent from water warmer than 25°C (Figs. 2 and 3). This result appears surprising given the short distances involved, but the observed pattern suggests that these frogs may have relatively small home ranges, or that the detectability and status of the disease changes rapidly with environmental conditions. Although other factors such as water chemistry may potentially affect *Bd* infection rates, our results strongly suggest a functional link between water temperature and *Bd* infection status in frogs.

Previous laboratory work conducted on other species indicates that amphibian hosts are able to clear *Bd* if infected individuals experience temperatures between 27–37°C. Caging great barred frogs (*Mixophyes fasciolatus*) at 27°C (n = 8) cured 50% of the frogs, which remained healthy, and tested negative for *Bd* when the experiment was terminated [7]. Orange-eyed treefrogs (*Litoria chloris*) cleared *Bd* within 16 hours after being caged at an environmental temperature of 37°C (n = 10) [12]. Western chorus frogs (*Pseudacris triserata*) collected in Arizona, cleared *Bd* following incubation at 32°C for 5 days (n = 6) [13]. American bullfrogs and northern cricket frogs (*Acris crepitans*) cleared *Bd* after being subjected to 30°C for 10 consecutive days, after which only one frog remained infected (n = 28) [14]. Heat treatments can also clear larval amphibians of *Bd*; 7 out of 8 tadpoles of the midwife toad (*Alytes obstetricans*) cleared *Bd* infection when exposed to temperatures higher than 26°C for 5 days [30]. Our results are consistent with these findings, despite having been conducted in uncontrolled environments. Indeed, the vast majority of *R. yavapaiensis* (51/52; 98%) captured in water warmer than 30°C were *Bd*− (Figs. 1A, [2], [3]). Overall, our results are consistent with the hypothesis that warm waters exclude *Bd* from infecting *R. yavapaiensis* hosts, although we cannot rule out all hypothetical alternatives.

In addition to the well-documented negative effects of warm waters on *Bd*, temperature is also strongly linked to amphibian immune system responses. Declines in amphibian immune defenses as temperatures decrease are well documented [31]–[34], which may explain why amphibians are particular susceptible to pathogens such as *Bd* that survive and grows at low temperatures [7], [10], [35]. Conversely, at warmer temperatures amphibians may be less susceptible to *Bd* and chytridiomycosis due to greater effectiveness of the immune response [36], [37]. Thermal impacts on *Bd*, and on the amphibian immune system have important ramifications for the ecology of chytridiomycosis and its impacts on wild amphibian populations [38].

We currently do not know if habitat choice is altered by a host's disease state, nor whether infected lowland leopard frogs deliberately seek out warmer sites to clear *Bd*. However, such speculation appears well supported, as diseased or parasitized amphibians and other ectotherms have been shown to actively seek temperatures above their thermal optima in order to generate ‘behavioral fevers’ that enhance host immune response or reduce pathogenic activity [12], [39], [40]; although see [41]. For example, green tree frogs (*Hyla cinerea*) behaviorally elevated their body temperature 2°C following inoculation with a pathogenic bacterium [42]. More directly, captive boreal toads (*Bufo* [*Anaxyrus*] *boreas*) with severe *Bd* infections shifted resting positions towards heat strips, suggesting a strategy to combat *Bd* infection [37]. Behavioral fever response to *Bd* has also been observed in wild populations. The average body temperature of a population of Panamanian golden frogs (*Atelopus zeteki*) increased 2.4°C following exposure to *Bd*, suggesting that the frogs exhibited a population-wide behavioral fever response during the epidemic [43]. The odds of *Bd* infection decreased with increasing body temperature, demonstrating that even slight environmental or behavioral changes have the potential to affect an individual's vulnerability to infection [43].

Since A.D. 79, when Pliny the Elder documented frogs inhabiting the hot springs of Pisa in his seminal work “Naturalis Historia”, amphibian populations have been observed in geothermal ecosystems around the world, including in Algeria [44], Taiwan [45], China [46], and Chile [47]. There are hundreds of geothermal watersheds throughout western North America, Central America, and Eastern Africa [48], many of which are located within the historic ranges of vulnerable species of amphibians [49]. Although geothermal ecosystems make up only a small fraction of most landscapes, they may be demographically important if they provide amphibians with even partial protection from temperature-sensitive diseases such as chytridiomycosis during pandemic events. Models suggest that the key to long-term persistence with *Bd* is survival of at least some fraction of infected adults–if some individuals survive the initial epidemic, it is possible that the infected amphibian population will persist in a new endemic state [50], [51].

Geothermal ecosystems may confer disease-protection to other amphibian species besides lowland leopard frogs. For example, in our study we sampled 20 individuals of other species in water temperatures ranging from 16–36°C (Table 1). Only two (10%) of these individuals were *Bd*+, and both individuals were captured at 16°C. Furthermore, geothermal ecosystems in Yellowstone National Park appear to be protecting boreal toads from redleg, a potentially fatal bacterial disease [52]. Finally, the relict leopard frog *Rana* (*Lithobates*) *onca*, a close relative to the lowland leopard frog [53], [54], is a rare species whose survival may be contingent on geothermal watersheds. The relict leopard frog was once thought to be extinct, but several populations were rediscovered in the 1990's, and all naturally occurring *R. onca* populations are now associated with perennial geothermal springs with source temperatures exceeding 30°C [18].

Elucidating the relationship between temperature and *Bd* prevalence has important implications for effective conservation, and reintroductions of threatened and endangered native amphibians [27]. No methods are currently available to treat amphibian populations against *Bd* in the wild; therefore susceptible species may persist only where conditions are not favorable for *Bd* or for chytridiomycosis outbreaks [24]–[26]. Our findings indicate that geothermal waters 25°C–37°C appear to provide amphibians with significant protection from *Bd* and, by extension, chytridiomycosis. While some species may not tolerate high temperatures, there is often a wide range of water temperatures present in geothermal ecosystems (Figs. 2 and 3). It may also be possible to experimentally augment temperatures in non-geothermal environments, thereby creating *Bd*-free microhabitats that can provide infected individuals with opportunities to clear themselves of the pathogen. Geothermal watersheds appear to represent habitats of exceptional conservation value for some amphibians, and emphasizing protection and restoration efforts, as well as native species translocations into suitable geothermal ecosystems could help recover threatened and endangered species.

## Materials and Methods

The research presented here was conducted in accordance with State University of New York, College of Environmental Science and Forestry Institutional Animal Care and Use Committee (IACUC) permit 2009-4 (amended), with Dr. Martin Schlaepfer as the Principal Investigator. The IACUC at the University of California San Diego also approved our Animal Use Protocol (Protocol Number S11013) on 1/31/2011, with Dr. Gregory Rouse as Principal Investigator.

We sampled amphibians from twelve sites in Arizona in 2004, 2009, and 2010 (Table 1). Seven of the sites were influenced by geothermal springs or wells, while five were not. We searched for frogs at night (except at Markham Creek, which was sampled by day). At each site, we captured as many individuals as possible by hand, using a new pair of disposable Nitrile gloves to capture and handle each animal. Each individual was retained in a new, closable plastic bag (Ziploc®) until all sampling was completed to ensure that animals were only sampled once, and to prevent cross-contamination. In order to minimize storage times, frogs were processed in the order of capture, generally within 30–90 min. No individuals showed obvious signs of stress, and all animals swam or hopped away immediately upon release.

*Bd* is transmitted aquatically [38], [55], [56]; therefore we used the water temperature at the place and time of each capture as an independent variable. This approach was intended to capture microhabitat (spatial) variation in water temperature, rather than relying on daily or monthly air temperatures at sites. It also has the benefit of being measured at the same time as the frog was sampled for *Bd*. When the captured frogs were partially or fully immersed in water, temperature was measured at the point of capture using a digital thermometer (CDN® Model Q2-450; accuracy ±0.5°C). Six frogs were captured on the banks (within less than 2 m of water), in which case the temperature was measured at the nearest water point. Samples from 2004 represent a subset of records from a previous survey of *Bd* in Arizona [20] that also included water temperature at the point of capture. We determined the sex (male, female, or juvenile) of captured frogs, and measured snout-vent length (SVL) and mass before releasing at the point of capture. We defined juveniles as individuals shorter than 50 mm SVL; the approximate size at which ranids in this group of species (“*pipiens*”) become sexually mature [57].

Methods for detecting the presence of *Bd* followed standard procedures. In 2009 and 2010 we used a Sterile Omni Swab (Whatman® WB100035) to sample skin cells from each animal's venter, flanks, and groin. We swabbed each amphibian a total of 25 times using the applicator, which was then ejected into in a 2-ml sterile tube filled with a buffer solution containing 50 mM Tris, pH 8, 50 mM EDTA, 25 mM Sucrose, 100 mM NaCl, and 1% SDS. In 2004, frogs were scraped 25 times in the same body locations using a wood applicator, which was then placed in 70% ethanol [20]. All samples were assayed within one month of being collected for the presence of *Bd* using Polymerase Chain Reaction amplification by a commercial lab (Pisces Molecular, Boulder, CO), following the methods from Annis et al [58] with modifications to increase sensitivity and specificity (J. Wood personal communication 2009). Experiments comparing skin scrapes versus skin swabs demonstrated that the ability to detect Bd-positive animals did not differ significantly between the methods [59]. Moreover, the Qiagen DNA spin column procedure that we used for DNA extraction is not inhibited by tannins or other compounds found in wood, unlike other DNA extraction methods (J. Wood, pers. comm., 2011).

**Figure 4:**
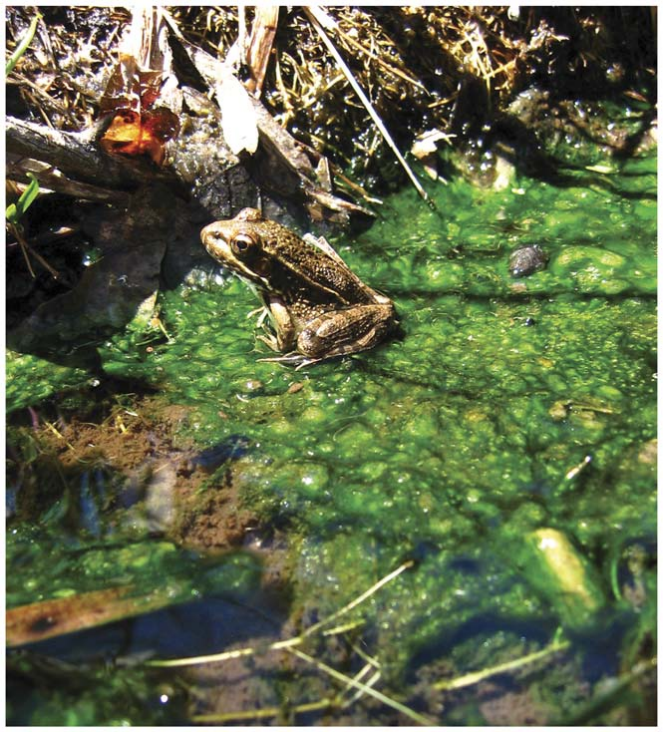
Lowland leopard frog in Muleshoe Hot Spring. Juvenile lowland leopard frog *Rana* (*Lithobates*) *yavapaiensis* inhabiting Muleshoe Hot Spring, a geothermal ecosystem near Willcox, Arizona. Frogs were repeatedly observed in waters 35–39°C during this study. (Photo credit: MA Schlaepfer).

We restricted our statistical analyses to the most common amphibian species, the lowland leopard frog (*Rana* [*Lithobates*] *yavapaiensis*) (Fig. 4). A binomial logistic regression using a logit link function tested for an association between the response variable (*Bd* presence/absence) and several predictor variables: Snout-Vent Length (SVL) of individual, Year, Distance to Solstice (absolute difference in days between the sampling date and June 21^st^ used to capture seasonal variation), Site, and water temperature. Two-sample t-tests, Fisher's exact, and chi-square tests were used to test for differences in distributions. Analyses were conducted in Minitab (vers. 15), and results are reported as means and standard errors.
